# Silencing *MdGH3-2/12* in apple reduces drought resistance by regulating AM colonization

**DOI:** 10.1038/s41438-021-00524-z

**Published:** 2021-04-01

**Authors:** Dong Huang, Qian Wang, Zhijun Zhang, Guangquan Jing, Mengnan Ma, Fengwang Ma, Chao Li

**Affiliations:** State Key Laboratory of Crop Stress Biology for Arid Areas/Shaanxi Key Laboratory of Apple, College of Horticulture, Northwest A & F University, Yangling, 712100 Shaanxi China

**Keywords:** Drought, Arbuscular mycorrhiza

## Abstract

Drought leads to reductions in plant growth and crop yields. Arbuscular mycorrhizal fungi (AMF), which form symbioses with the roots of the most important crop species, alleviate drought stress in plants. In the present work, we identified 14 *GH3* genes in apple (*Malus domestica*) and provided evidence that *MdGH3-2* and *MdGH3-12* play important roles during AM symbiosis. The expression of both *MdGH3-2* and *MdGH3-12* was upregulated during mycorrhization, and the silencing of *MdGH3-2*/*12* had a negative impact on AM colonization. *MdGH3-2/12* silencing resulted in the downregulation of five genes involved in strigolactone synthesis, and there was a corresponding change in root strigolactone content. Furthermore, we observed lower root dry weights in RNAi lines under AM inoculation conditions. Mycorrhizal transgenic plants showed greater sensitivity to drought stress than WT, as indicated by their higher relative electrolytic leakage and lower relative water contents, osmotic adjustment ability, ROS scavenging ability, photosynthetic capacity, chlorophyll fluorescence values, and abscisic acid contents. Taken together, these data demonstrate that *MdGH3-2*/*12* plays an important role in AM symbiosis and drought stress tolerance in apple.

## Introduction

To cope with a variety of environmental challenges, plants have evolved many strategies, including interactions with other microorganisms^1^. Arbuscular mycorrhizal (AM) symbiosis is one of the most complex and beneficial interrelationships and is formed between the roots of nearly 80% of terrestrial plants and fungi from the phylum Glomeromycota^2–4^. This symbiosis evolved more than 450 million years ago, making it one of the most ancient symbioses, and it provides a significant selective advantage to both symbiotic partners^5^. After a molecular dialog between the two partners, the AM fungus penetrates the host roots and forms branched structures in cortical cells in which nutrient exchange occurs^6^. As obligate symbionts, AM fungi (AMF) complete their life cycle using the carbon sources provided by their host plants. In exchange, fungi not only improve the availability of mineral nutrients (especially phosphate) for the partner but also enhance its resistance to various environmental stresses^2,7,8^.

The establishment of AM symbiosis is a complex process that requires molecular communication between the two partner organisms^9,10^. Over the past decade, great progress has been made in understanding how AM symbiosis is established^11–13^. The process of symbiosis formation is relatively consistent between different plant and AMF species and can be divided into several stages. First, before coming into contact with the AMF, the plant roots secrete strigolactone (SL) “branching factors”. These branching factors stimulate the spores of AM fungi germination and branching, which continue to approach the roots^14^. At the same time, AMF also secrete signaling substances called “mycorrhizal factors”, which include sulfated and nonsulfated lipochitooligosaccharides^15,16^. After the plant roots recognize these substances, the host plants activate common symbiotic signaling pathways^17^. When the fungal mycelium comes into contact with the plant root epidermal cells, it differentiates into an attachment structure called a hyphopodium, and the root epidermal cells also undergo cytoskeletal changes. The fungal hyphae penetrate the epidermal cells and continue to elongate toward the cortical cells, continuously growing through the root tissue^18^. When the mycelium extends to the endothelial layer of cells and penetrates the cell wall, it continues to branch in the cells, forming a highly branched, tree-like structure that becomes an arbuscule. Arbuscules are the main site of the exchange of mineral nutrients between the host cell and the fungi^12,13^.

Plant hormones are multifunctional molecules that act as central regulators of all plant developmental processes, including the development of the plant-AMF interaction^12,13,19–22^. Several studies have reported that different hormones are involved in AM formation^12,20,23,24^, and auxin and its derivatives are thought to play a particularly important role. Hanlon and Coenen^25^ showed that the roots of two auxin-related mutants were poorly colonized. Similarly, a low-auxin *bsh* mutant of pea (*Pisum sativum*) exhibited a reduction in mycorrhizal colonization and strigolactone exudation^26^. Guillotin et al.^9^ showed that *Sl-IAA27* may control SL biosynthesis via the regulation of *NSP1*. Although all plant hormones can perform their biological functions independently, the regulation of a plant’s development throughout its lifespan requires the synergistic action of multiple hormones^24^.

In agricultural ecosystems, drought is one of the most serious environmental factors that limit plant growth and crop productivity^27,28^. AM symbiosis can improve the performance of plants under various environmental stresses, such as drought, and can alter plant water relations under conditions of both water sufficiency and water stress^3,29–31^. The regulation of plant drought resistance by AMF is a very complex process involving many metabolites and metabolic pathways^32,33^. AMF symbiosis has been shown to improve plant drought adaptability by up- and downregulating many physiological and biochemical processes. Specifically, AMF can directly promote the absorption and transport of plant nutrients and water, increase plant osmotic regulation, improve plant gas exchange capacity and water use efficiency, and increase plant antioxidant capacity^4,30,34^.

In this study, the expression of two apple early auxin-responsive genes, *MdGH3-2* (MD02G1180300) and *MdGH3-12* (MD15G1290900), was shown to be significantly induced by AM fungi. To further understand their potential roles in the mycorrhizal process, an RNA interference (RNAi) approach was used to repress their expression. We hypothesized that their repression would not only affect mycorrhization but would also affect drought resistance in apple under drought conditions. Physiological parameters such as photosynthetic parameters, antioxidant system activity, osmotic adjustment, and ABA levels under AM inoculation were assessed. Overall, the data show that *MdGH3-2/12* are involved in AM symbiosis and drought stress tolerance in apple.

## Results

### Identification of *MdGH3* genes

In total, fifteen *MdGH3* family genes were identified. However, we found that there was one gene (MD09G1091000) with 280 amino acids, and the predicted conserved domain indicated that it was incomplete. Therefore, this gene was not analyzed further. The basic information for the *MdGH3* genes, including gene names, gene IDs, chromosome locations, protein lengths, theoretical isoelectric points (pIs), and molecular weights (MWs), is listed in Table 1. The sizes of the MdGH3 proteins ranged from 562–709 amino acids. The MW and pI of the MdGH3 proteins ranged from 62531.86 Da (MdGH3-7) to 80113.10 Da (MdGH3-3) and from 5.41 (MdGH3-11) to 7.54 (MdGH3-3), respectively.

**Table 1:** The *GH3* family in apple

Gene name	Gene ID (GDDH13)	Gene ID (V1.0)	Chromosome location	Length (aa)	MW (Da)	pI
MdGH3-1	MD01G1076500	MDP0000233483	Chr01:18316873..18322302	562	62595.79	6.01
MdGH3-2	MD02G1180300	MDP0000612660	Chr02:16058335..16061924	599	67466.49	6.23
MdGH3-3	MD03G1215800	MDP0000132162	Chr03:29660247..29662938	709	80113.10	7.54
MdGH3-4	MD03G1284700	MDP0000568498	Chr03:36483028..36485639	596	67617.14	6.43
MdGH3-5	MD05G1092900	MDP0000209432	Chr05:19753185..19755519	601	67564.48	5.92
MdGH3-6	MD05G1092300	MDP0000873893	Chr05:19618797..19621192	577	64773.60	6.30
MdGH3-7	MD07G1145900	MDP0000786650	Chr07:21291062..21294030	562	62531.86	6.22
MdGH3-8	MD11G1293000	MDP0000231245	Chr11:41253547..41255976	575	64085.78	5.49
MdGH3-9	MD11G1304200	MDP0000204381	Chr11:41930461..41932991	596	67407.79	5.87
MdGH3-10	MD11G1230400	MDP0000402444	Chr11:33501665..33504059	614	69316.51	7.03
MdGH3-11	MD13G1132300	MDP0000226842	Chr13:10035347..10038041	611	69630.77	5.41
MdGH3-12	MD15G1290900	MDP0000872868	Chr15:26870302..26873401	599	67351.40	5.45
MdGH3-13	MD16G1142800	MDP0000834656	Chr16:10976308..10979216	611	69665.62	5.53
MdGH3-14	MD17G1081000	MDP0000214081	Chr17:6765282..6769196	613	69505.96	6.85

### Phylogenetic and gene structure analysis

**Fig. 1:**
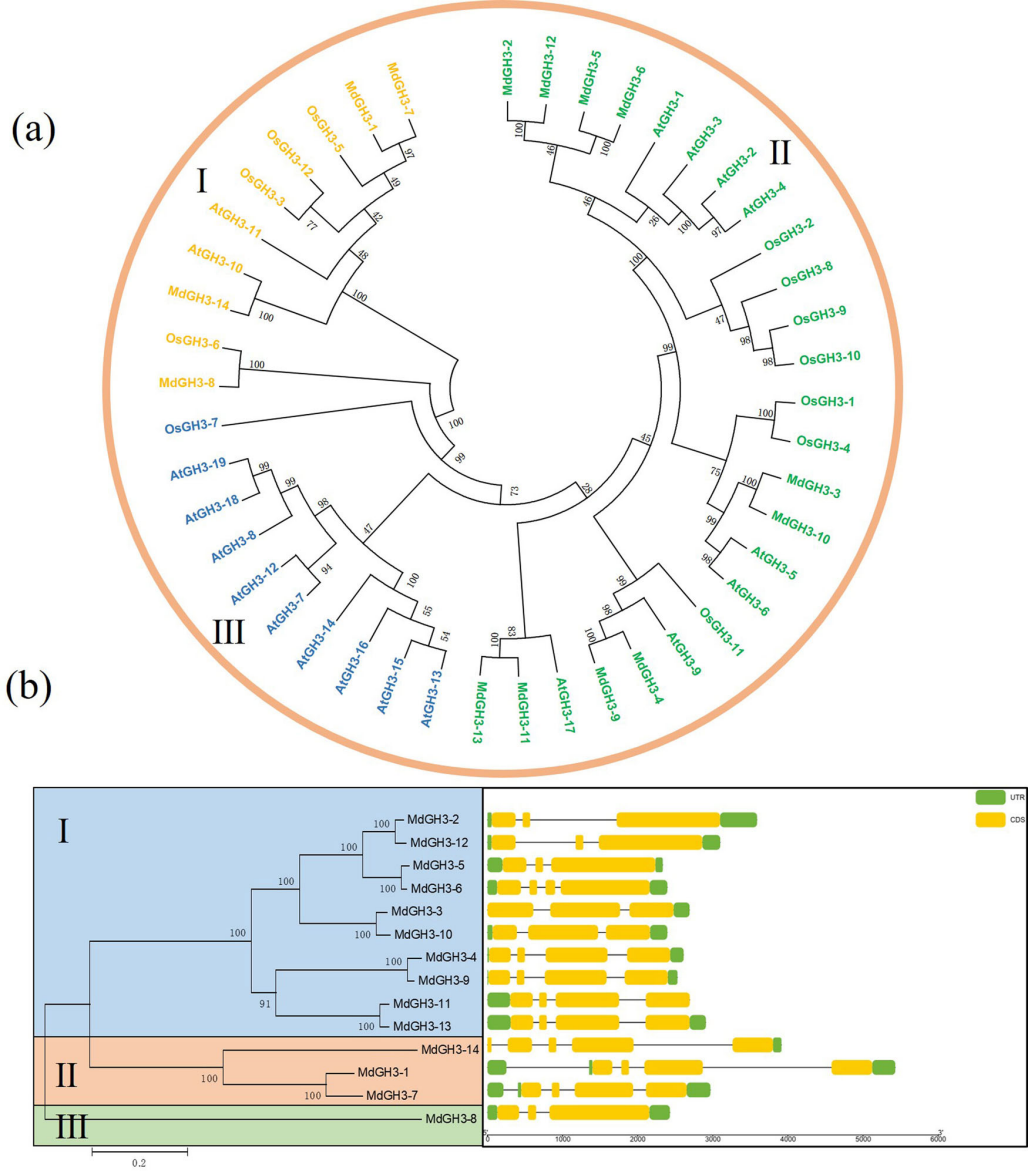
Phylogenetic analysis of GH3 proteins from apple, *Arabidopsis*, and rice and gene structures of *MdGH3* genes. **a** The phylogenetic tree was constructed with the MEGA5 program using the neighbor-joining method. **b** Gene structures were constructed using TBtools

To investigate the evolutionary relationships among apple, *Arabidopsis* (*Arabidopsis thaliana*), and rice (*Oryza sativa* L.) GH3s proteins, we constructed a phylogenetic tree (Fig. 1a). The GH3 members could be classified into three subgroups, numbered I, II, and III. There were 10, 25, and 10 GH3s in the three subgroups, respectively. Fourteen *MdGH3s* were unevenly distributed among three different subgroups. The gene structure analysis showed that in group I, *MdGH3-2*, *MdGH3-3*, *MdGH3-5, MdGH3-10*, and *MdGH3-12* contained three exons, and the other genes contained four exons. In group II, *MdGH3-14* contained five exons, and *MdGH3-1* and *MdGH3-7* contained four exons. In group III, *MdGH3-8* had three exons. Even within the same phylogenetic subgroup, the *MdGH3* members exhibited different structural patterns of exon–intron junctions (Fig. 1b).

### Expression and subcellular localization of MdGH3-2 and MdGH3-12

**Fig. 2:**
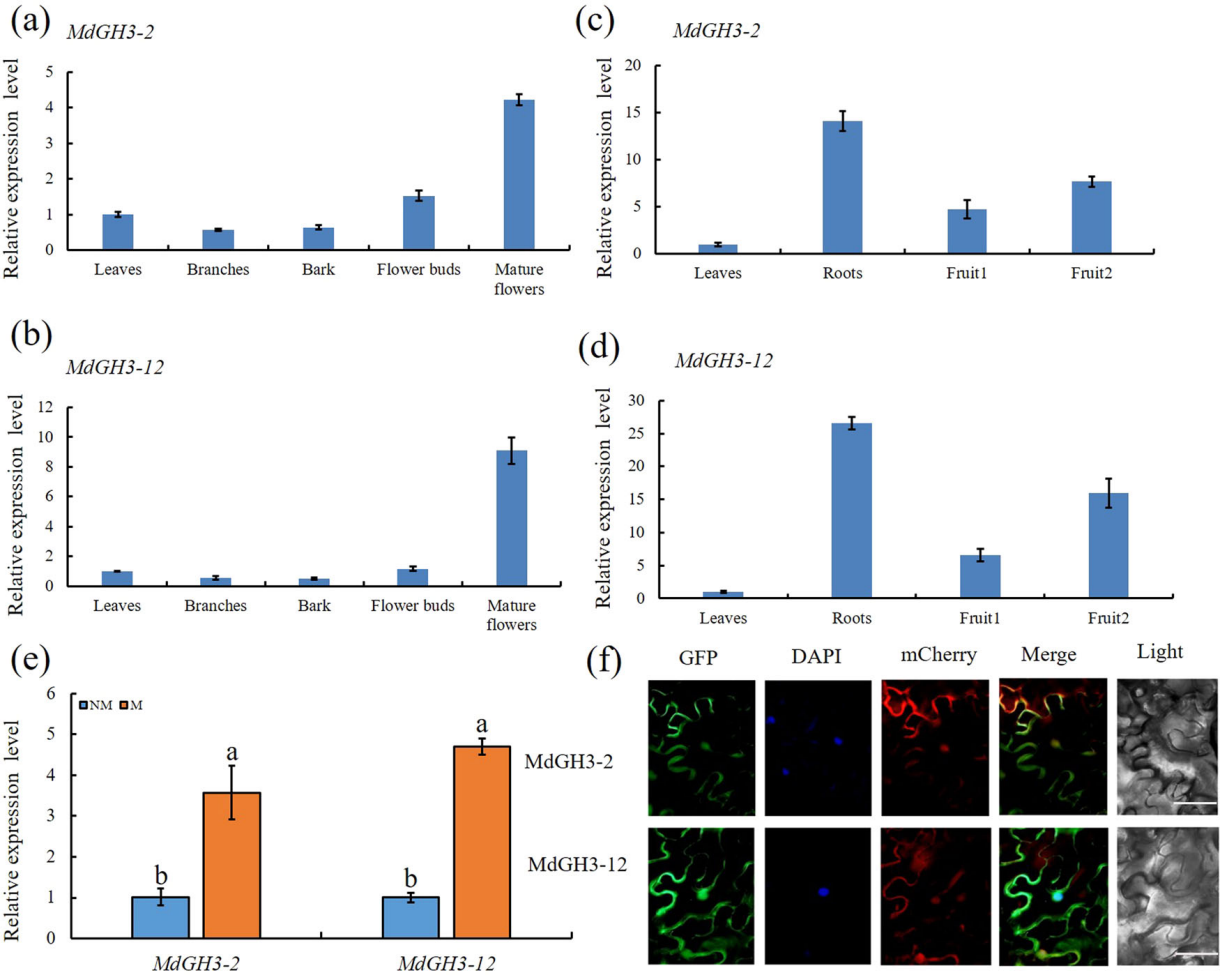
*MdGH3-2* and *MdGH3-12* expression in various tissues, response to AM colonization, and protein localization. **a** Expression profiles of *MdGH3-2* in ‘Royal Gala’ (*Malus domestica*). **b** Expression profiles of *MdGH3-12* in ‘Royal Gala’ (*Malus domestica*). **c** Expression profiles of *MdGH3-2* in ‘Winter Red’. **d** Expression profiles of *MdGH3-12* in ‘Winter Red’. **e** Expression of *MdGH3-2* and *MdGH3-12* in M and NM apple roots inoculated with *Rhizophagus irregularis* over 8 weeks. **f** MdGH3-2 or MdGH3-12 localized in the nucleus and membranes of tobacco leaf epidermal cells. AtCBL1n-mCherry served as a marker for plasma membrane protein localization. M=mycorrhizal, NM=nonmycorrhizal. Fruit1=4 months after anthesis, Fruit2 = 7 months after anthesis. Bars = 50 μm. Data are expressed as means ± SDs (*n* = 3). Data with different letters differ significantly (*P* < 0.05), as determined by the independent-samples t-test

The expression of *MdGH3-2* and *MdGH3-12* differed among plant organs. In ‘Royal Gala’, *MdGH3-2*, and *MdGH3-12* were highly expressed in mature flowers, but their expression levels were lower in branches and bark (Fig. 2a, b). In ‘Winter Red’, *MdGH3-2* and *MdGH3-12* were highly expressed in roots (Fig. 2c, d). When we investigated the expression of *MdGH3s* after inoculation with *Rhizophagus irregularis*, we found that *MdGH3-2* and *MdGH3-12* were significantly induced in mycorrhizal plants compared with their expression in nonmycorrhizal (NM) plants (Fig. 2e, Fig. S1). Furthermore, subcellular localization analysis of *MdGH3-2* and *MdGH3-12* showed that their encoded proteins were localized to membranes and nuclei (Fig. 2f). The nucleotide sequences of *MdGH3-2* and *MdGH3-12* were highly (up to 90%) similar (Fig. S2), and it was therefore not possible to design primers that specifically knocked down *MdGH3-2* or *MdGH3-12*. The two genes could only be silenced simultaneously.

### *MdGH3-2* and *MdGH3-12* are positive regulators of mycorrhization

**Fig. 3:**
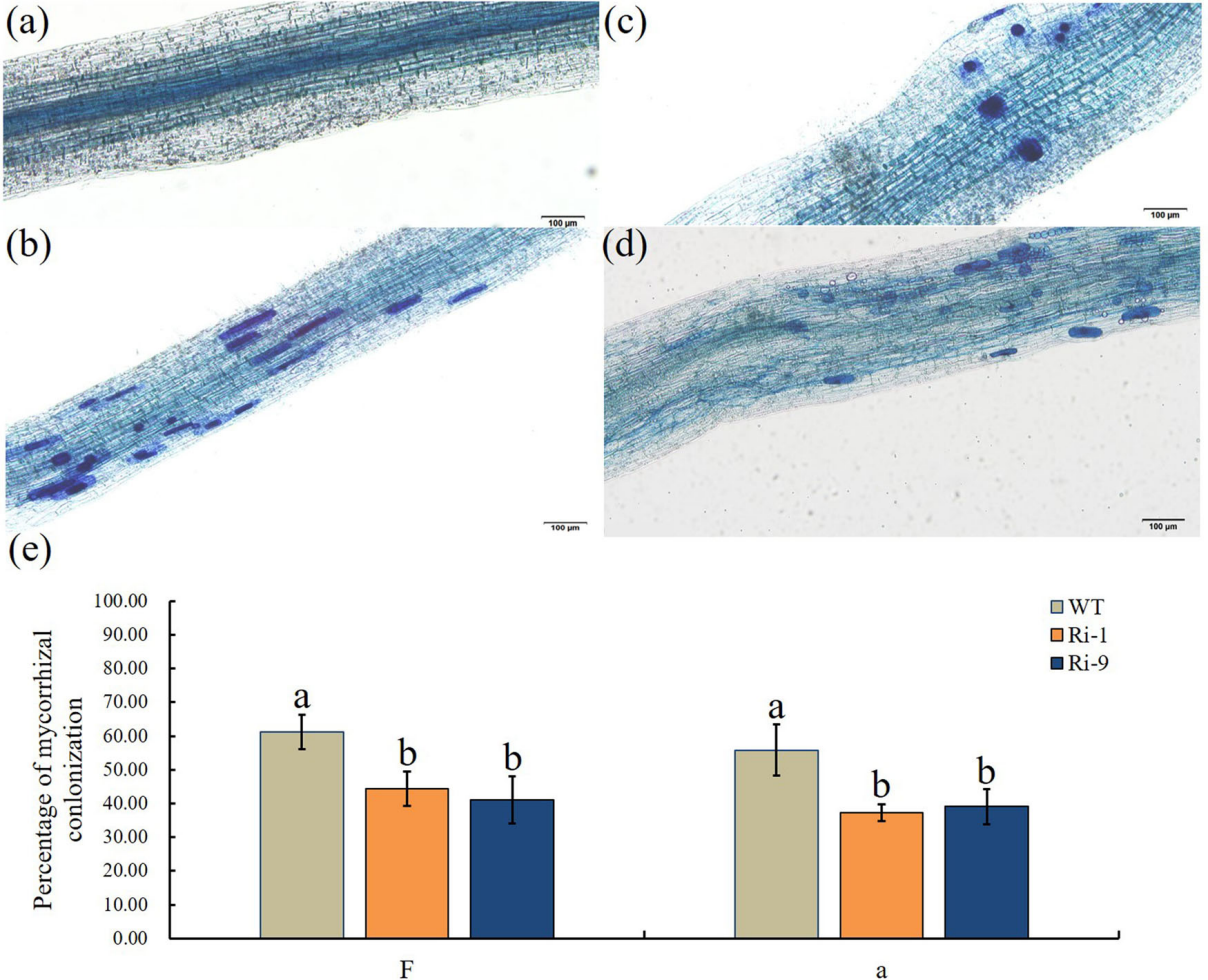
Mycorrhizal phenotype of RNAi lines and WT 8 weeks after inoculation with *Rhizophagus irregularis*. **a**–**d** Root segments stained with trypan blue. **a** Roots of WT under the NM condition. **b**–**d** Arbuscules of WT, Ri-1 and Ri-9. **e** Colonization percentages in the RNAi lines and WT. ‘F’, frequency of colonization in the root system; ‘a’, arbuscule abundance (percentage) in the colonized root sections. Bars = 100 μm. Data are expressed as means ± SDs (*n* = 3). Data with different letters differ significantly (*P* < 0.05), as determined by one-way ANOVA and Tukey’s test

We next investigated whether *MdGH3-2/12* plays a role during mycorrhization. We obtained two individual RNAi lines and used genome walking to identify the T-DNA integration site. The results showed that their insertion sites were located on two different chromosomes (Table S1). The expression levels of *MdGH3-2* in lines Ri-1 and Ri-9 were reduced by 36% and 25%, respectively. The expression levels of *MdGH3-12* in lines Ri-1 and Ri-9 were reduced by 30% and 28%, respectively (Fig. S3). We also detected the expression levels of other *GH3* family genes in the two transgenic lines. The results showed that other genes were not affected by the silencing of *MdGH3-2/12* (Fig. S4). The two RNAi lines and wild-type (WT) plants were inoculated with *R. irregularis*. After 8 weeks, symbiotic relationships had formed in both the WT and RNAi lines (Fig. 3b–d). However, the frequency of colonization (F) and arbuscule abundance (a) in the two RNAi lines were significantly lower than those in WT (Fig. 3e). Taken together, these data indicated that *MdGH3-2/12* were involved in AM symbiosis.

### Expression of strigolactone biosynthesis genes and strigolactone production

**Fig. 4:**
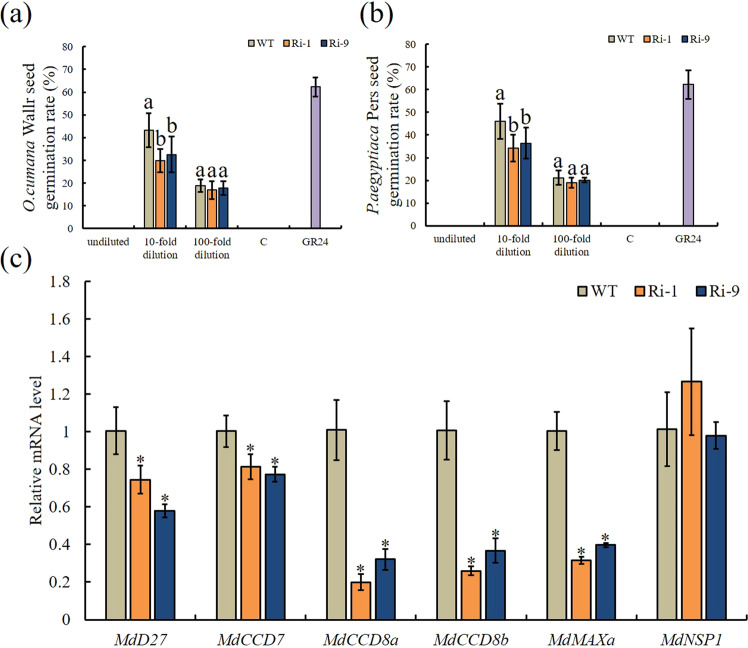
Quantification of strigolactones and expression of strigolactone biosynthesis genes in roots of WT and RNAi lines without mycorrhization. Germination of *Orobanche cumana* Wallr. **a** and *Orobanche aegyptiaca* Pers. **b** seeds in response to a range of concentrations of root exudates of WT and RNAi lines. Sterile water (C) and 1 mg L^-1^ synthetic strigolactone (GR24) were used as negative and positive controls. **c** Expression of strigolactone biosynthetic genes and *MdNSP1* in WT and RNAi lines under NM conditions. NM=nonmycorrhizal. Data are expressed as means ± SDs (*n* = 4 for **c**, *n* = 12 for **a** and **b**). Data with different letters and asterisks differ significantly (*P* < 0.05), as determined by one-way ANOVA and Tukey’s test

Because SL plays important role during AM symbiosis, we also analyzed SL production. To quantify SL production in the WT and RNAi lines, we performed a germination bioassay with seeds of *Orobanche cumana* Wallr. and *Orobanche aegyptiaca* Pers. using root extracts from the NM WT and RNAi lines (Fig. 4a, b). As expected, the seed germination rates were null when the seeds were treated with sterile distilled water. However, when treated with GR24, the germination rates of the *O*. *cumana* and *O*. *aegyptiaca* seeds were 62.30% and 62.22%, respectively. Furthermore, significantly reduced germination rates were obtained when both seed species were treated with RNAi-line root extracts at a 10-fold dilution. The germination rates of the seeds after treatment with the WT and RNAi extracts at a 100-fold dilution did not differ significantly, but the germination rates of the seeds treated with the RNAi line extracts were slightly lower than those of the seeds treated with the WT extracts. As a second approach to analyzing SL production, the expression patterns of SL biosynthetic pathway genes were analyzed (Fig. 4c). The results showed that the expression of those genes in NM RNAi lines was significantly lower than that in NM WT plants. The expression of *MdNSP1*, an important *GRAS* transcription factor in the nodulation process that is involved in AM symbiosis and regulates the expression of *D27* and *MAXa*, did not differ significantly among genotypes.

### Morphometric data on plant biomass

**Fig. 5:**
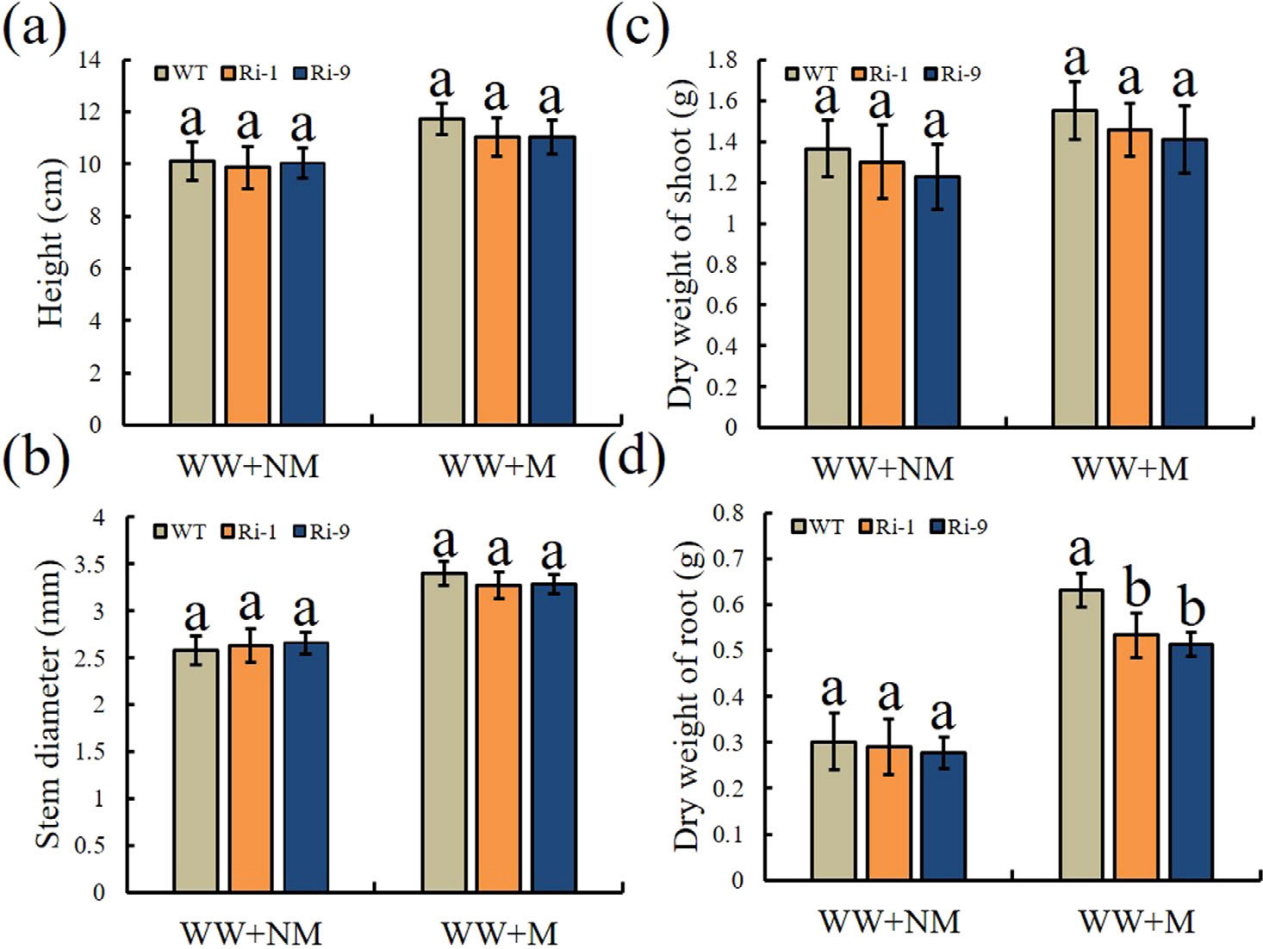
Influence of AM symbiosis on plant growth parameters and biomass production in WT and RNAi lines. **a** Plant height. **b** Stem diameter. **c**, **d** Shoot and root dry weight. M = mycorrhizal, NM = nonmycorrhizal. Data are expressed as means ± SDs (*n* = 5 for **c** and **d**, *n* = 10 for **a**, **b**). Data with different letters differ significantly (*P* < 0.05), as determined by one-way ANOVA and Tukey’s test

Growth promotion in host plants is the most direct effect of AMF inoculation. We, therefore, measured plant growth parameters and biomass production prior to the DS treatment. As expected, the plants inoculated with *R. irregularis* consistently exhibited greater plant growth and biomass production than the NM plants (Fig. 5a–d). Under WW+NM conditions, the plant height, stem diameter, and shoot and root dry weight did not differ significantly among genotypes. This trend was maintained under WW+M conditions, with the exception of root dry weight, which was significantly lower in Ri-1 and Ri-9 than in WT.

### RNAi*-MdGH3-2/12* plants are more sensitive to drought stress

**Fig. 6:**
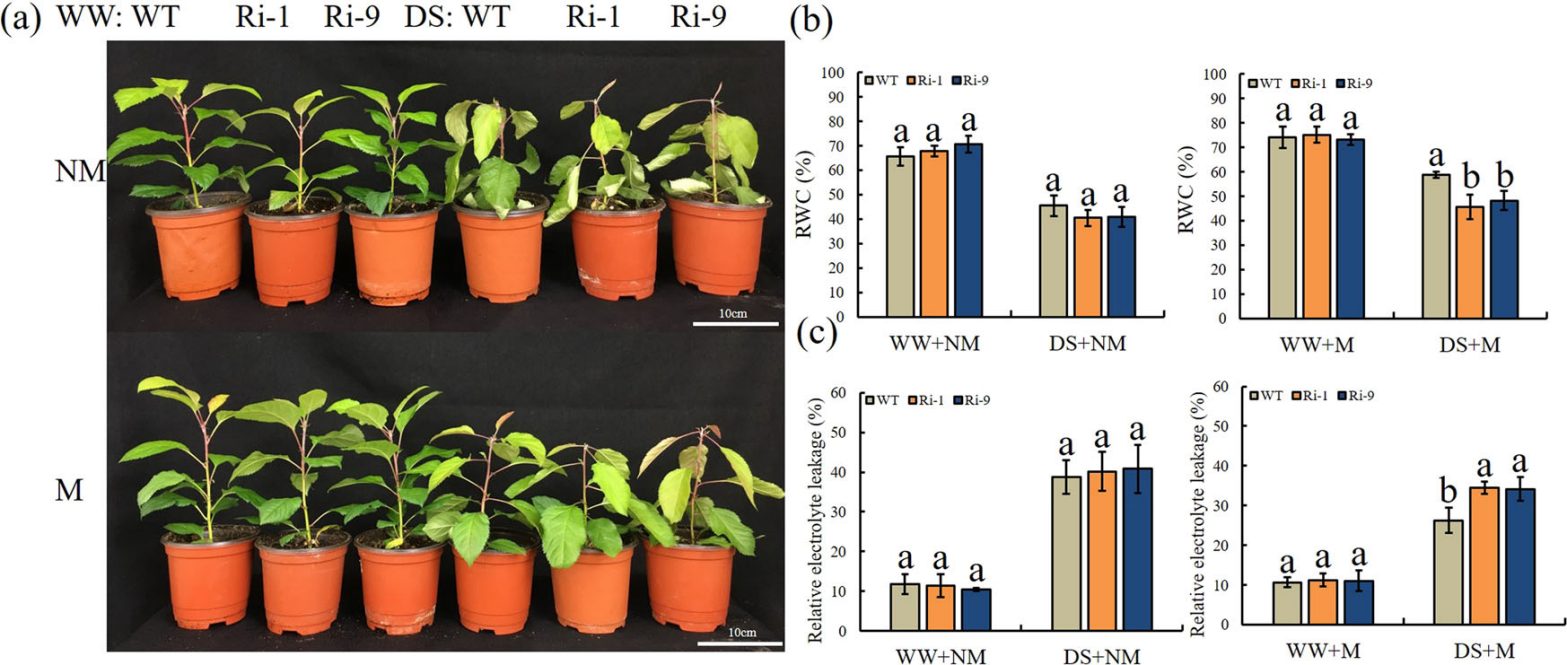
Drought tolerance in RNAi lines and WT. **a** Drought tolerance in RNAi lines and WT under M and NM conditions. **b** Relative water content (RWC). **c** Relative electrolyte leakage (REL). WW = well-watered, DS = drought stress, M = mycorrhizal, NM = nonmycorrhizal. Data are expressed as means ± SDs (*n* = 3). Data with different letters differ significantly (*P* < 0.05), as determined by one-way ANOVA and Tukey’s test

RNAi*-MdGH3-2/12* transgenic lines with reduced *MdGH3-2* and *MdGH3-12* expression had lower root SL content and lower levels of AMF colonization. We have previously shown that AM fungi enhance drought resistance in apple. Therefore, we wanted to assess whether repression of both *MdGH3-2* and *MdGH3-12* would affect the performance of apple under drought conditions because of lower AM colonization levels. Under NM and WW+M conditions, the phenotypes did not differ significantly among genotypes. However, under DS+M conditions, the RNAi lines showed more wilting and necrosis than the WT (Fig. 6a). This phenotype was also reflected in the REL and RWC values. Under the NM and WW+M treatments, there were no significant differences in RWC and REL among genotypes. However, under the DS+M treatment, the RWC was significantly lower in Ri-1 and Ri-9, at 77.50% and 82.13% that of WT, respectively (Fig. 6b). The REL was significantly higher in Ri-1 and Ri-9, at 1.31 and 1.30 times that of WT (Fig. 6c).

### Gas exchange and F*v*/F*m*

**Fig. 7:**
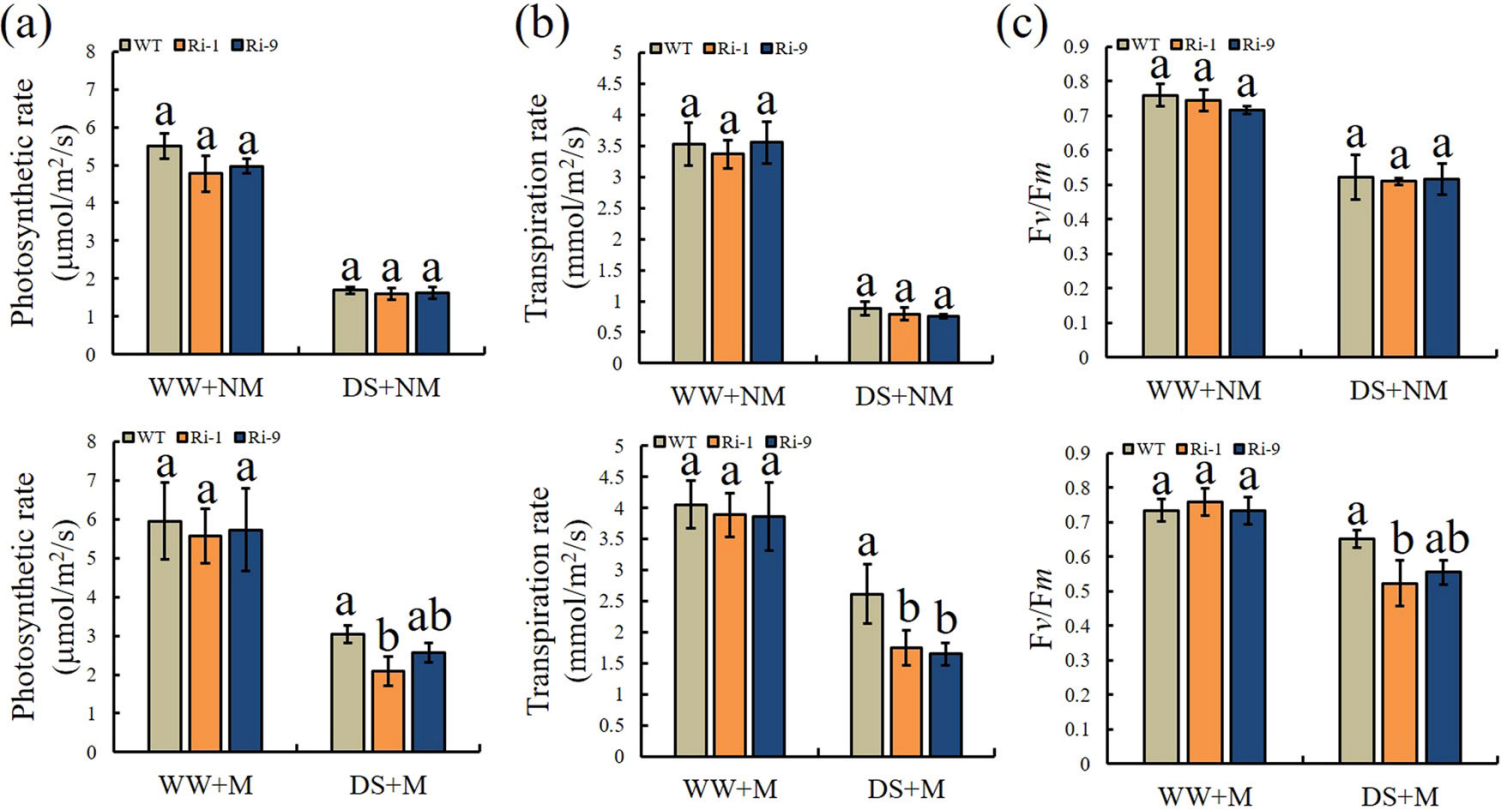
Effect of drought and AM symbiosis on Pn, Tr, and F*v*/F*m* in the RNAi lines and WT. **a** Photosynthetic rate (Pn). **b** Transpiration rate (Tr). **c** Maximum photochemical efficiency of PSII (F*v*/F*m*). WW=well-watered, DS=drought stress, M=mycorrhizal, NM=nonmycorrhizal. Data are expressed as means ± SDs (*n* = 3). Data with different letters differ significantly (*P* < 0.05), as determined by one-way ANOVA and Tukey’s test

Under the NM treatments, the values of Pn, Tr, and F*v*/F*m* were similar in the WT and RNAi lines, and the differences among them were not significant (Fig. 7). The results were similar for Pn, Tr, and F*v*/F*m* under the WW+M treatment. However, under the DS+M treatment, the values of Pn, Tr, and F*v*/F*m* were higher in the WT than in the RNAi lines. The Pn of WT was 1.46 and 1.18 times those of Ri-1 and Ri-9, respectively. Tr was significantly lower in Ri-1 and Ri-9 than in WT, at 66.79% and 63.03% that of WT, respectively. The same pattern was observed for F*v*/F*m*, and the Ri-1 and Ri-9 values were 80.27% and 85.12% that of WT, respectively.

### H_2_O_2_ and O_2_^-^ generation, antioxidant enzyme activity, and proline contents

**Fig. 8:**
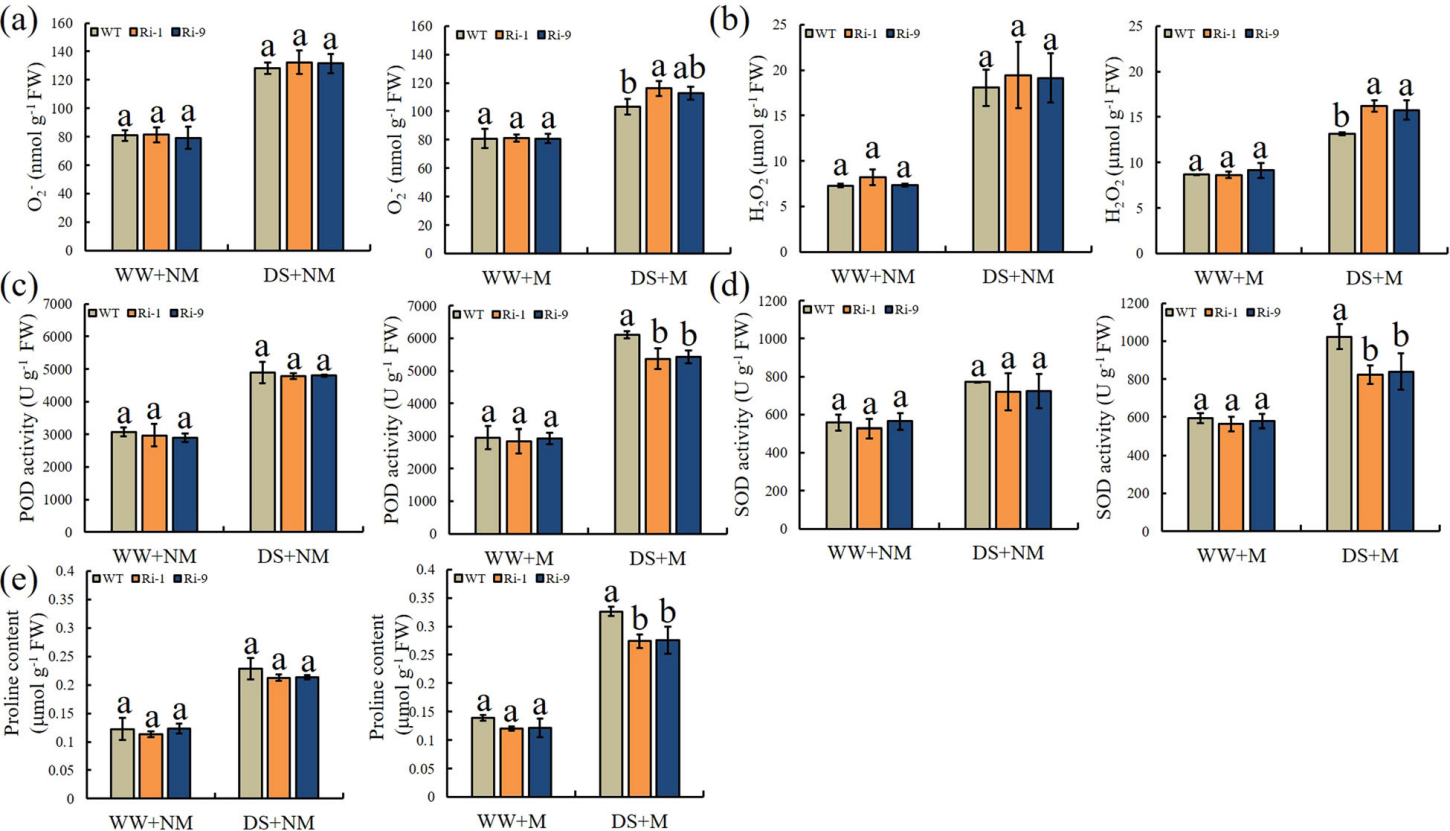
Effect of drought and AM symbiosis on the levels of ROS, antioxidant enzyme activity, and proline in WT and RNAi lines. Concentrations of **a** O_2_^-^ and **b** H_2_O_2_, **c**, **d** peroxidase (POD) and superoxide dismutase (SOD) activity, and **e** proline content. WW=well-watered, DS=drought stress, M=mycorrhizal, NM=nonmycorrhizal. Data are expressed as means ± SDs (*n* = 3). Data with different letters differ significantly (*P* < 0.05), as determined by one-way ANOVA and Tukey’s test

The drought treatment increased the O_2_^-^ and H_2_O_2_ concentrations in all plant types. Lower amounts of O_2_^-^ and H_2_O_2_ were detected in the WT than in the RNAi lines under DS+M (Fig. 8a, b). The O_2_^-^ concentration in Ri-1 was significantly increased, by 1.12 times, relative to that in WT, and the H_2_O_2_ concentrations in Ri-1 and Ri-9 were significantly increased, by 1.23 and 1.20 times, relative to that in WT, respectively. POD and SOD activities did not differ among genotypes under the NM and WW+M treatments (Fig. 8c, d). Under DS+M, the POD activities of Ri-1 and Ri-9 were significantly decreased, to 88.02% and 88.82% of the WT level, respectively. The SOD activities of Ri-1 and Ri-9 were significantly decreased, to 80.53% and 82.05% of WT levels, respectively. A similar pattern was observed for the proline content (Fig. 8e). Under DS+M conditions, the proline contents of Ri-1 and Ri-9 were significantly decreased, to 84.07% and 84.48% of WT levels, respectively. Together, these findings suggest that the RNAi lines exhibited reduced antioxidant enzyme activity and lower osmoprotectant contents under DS+M conditions than WT.

### ABA accumulation

**Fig. 9:**
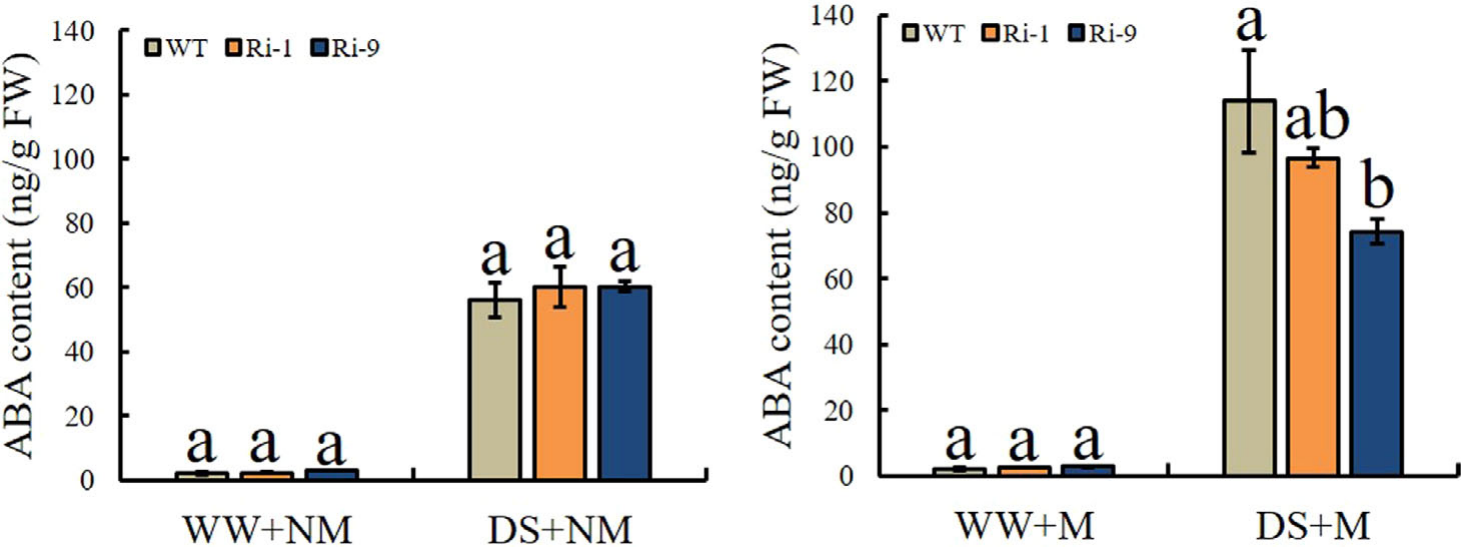
Effect of drought and AM symbiosis on root abscisic acid (ABA) content in WT and RNAi lines. WW = well-watered, DS = drought stress, M = mycorrhizal, NM = nonmycorrhizal. Data are expressed as means ± SDs (*n* = 3). Data with different letters differ significantly (*P* < 0.05), as determined by one-way ANOVA and Tukey’s test

Abscisic acid is a plant hormone that is very important to plant growth and development. It is usually related to plant responses to abiotic stresses such as drought. ABA is also related to AM symbiosis. As expected, drought enhanced the accumulation of ABA, especially under M conditions (Fig. 9). The data also showed that under the DS+M treatment, ABA accumulation in the WT was 1.18 and 1.53 times those in Ri-1 and Ri-9, respectively, and was significantly higher than that in Ri-9, reaching 113.94 ng g^-1^ FW.

## Discussion

As global temperatures increase, soil water shortages often occur^35^, and the resulting drought is one of the main factors limiting crop production worldwide^28^. In the process of evolution, plants have evolved mechanisms to flexibly adapt to adverse conditions^36^. One of these strategies is the establishment of AM symbiosis^4,37^. It is generally believed that this symbiotic relationship is a key component of plants’ ability to cope with drought^38^. Our previous research showed that AMF can directly increase plant osmotic regulation, improve plant gas exchange capacity and water use efficiency, and improve plant antioxidant capacity under drought stress^39^. We hypothesized that changes in the AMF colonization level would affect plant growth and alter plant performance under drought.

Many studies have shown that plant hormones, as signaling molecules, are involved in the regulation of AM symbiosis^19^. Moreover, interactions among different hormones regulate various plant physiological processes, as well as interactions between plants and microorganisms. Many auxin-responsive genes play roles in the regulation of mycorrhization. One example of this is the tomato *GH3* family member *GH3.4*, whose expression is highly correlated with mycorrhizal colonization levels^40^. The auxin amidohydrolases *MtIAR33* and *MtIAR34* are significantly upregulated by inoculation with AM fungi^41^. Another study showed that the positive effect of ABA on AM colonization was mediated mainly through *PP2AB'1*^12^. In our study, we identified 14 *GH3* genes in apple, and *MdGH3-2* and *MdGH3-12* were significantly induced by mycorrhizal inoculation. We used an RNAi approach to repress the expression of both *MdGH3-2* and *MdGH3-12* to gain insight into their functions during AM symbiosis and ultimately determine how they affect apple performance under drought stress. Interestingly, the expression levels of five SL synthesis genes were downregulated in the roots of the two transgenic lines relative to those in the WT. We also measured *MdNSP1* expression, and no significant differences were observed between RNAi lines and WT. These results suggest that *MdGH3-2/12* do not affect *MdD27* and *MdMAXa* expression through the regulation of *NSP1*. Moreover, a germination assay using two types of *Orobanche* seeds demonstrated that the root SL contents of the RNAi lines were lower than that of WT. We also observed a lower root AMF colonization frequency and arbuscule abundance in the two transgenic lines. These data suggest that *MdGH3-2* and *MdGH3-12* regulate SL biosynthesis and mycorrhization in apple.

A large body of research has documented the beneficial effects of different AM fungi on plant growth^30,42^. Here, we also showed that mycorrhizal symbiosis improved plant growth performance and increased plant biomass. In addition to the lower AMF colonization levels in the two RNAi lines, we also observed lower root dry weights in the Ri-1 and Ri-9 lines under WW+M conditions relative to that in WT. These results indicate that higher levels of AMF colonization improved the growth of mycorrhizal plants. Drought stress negatively affects plant growth and causes a series of physiological, biochemical, and molecular changes^32^. A large number of studies have reported that AM fungi enhance the ability of plants to resist drought stress through multiple mechanisms: improving the soil structure, promoting the formation of soil aggregates, and increasing soil nutrient and moisture retention; accelerating plant nutrient and water absorption through the activity of extraradical hyphae; improving plant photosynthetic function; increasing the efficiency and capacity of the antioxidant system to alleviate harmful ROS effects, and increasing the expression of drought resistance genes^29,30^. Here, we were interested in determining whether lower AMF colonization levels in transgenic apples would increase their sensitivity to drought stress.

Drought stress can lead to a decrease in plant RWC, which is an important index for the evaluation of plant water status^43^. We found that under DS+M, RWC was significantly lower in the two RNAi lines than in WT. REL is an important indicator for evaluating membrane integrity and the extent of tolerance to abiotic stress^44^. Here, REL was significantly lower in WT than in the two RNAi lines under DS+M conditions. These data indicated that silencing *MdGH3-2/12* led to greater cell damage under DS+M conditions. Increased ROS production is a typical physiological response induced by drought stress^44^. High levels of ROS accumulation are harmful to cells and eventually lead to oxidative stress injury. Here, the RNAi lines accumulated more H_2_O_2_ and O_2_^-^ than the WT under DS+M. To maintain the normal physiological and metabolic functions of cells under stress, plants have developed a complete set of ROS scavenging defenses over the course of their long-term evolution, including enzymatic defenses such as SOD and POD. Multiple studies have shown that AM symbiosis increases the activity of antioxidant enzymes under drought stress^29,30^. Likewise, we found that POD and SOD activities were significantly lower in transgenic plants under the DS+M treatment than in WT. This result was also consistent with the measured contents of H_2_O_2_ and O_2_^-^ in the plants.

Mycorrhizal seedlings usually have higher photosynthetic rates than nonmycorrhizal seedlings under drought stress^39^. ROS produced by abiotic stress can damage components of the photosynthetic system and inhibit the repair of PSII. Plants can reduce ROS accumulation and damage to the photosynthetic system by inducing the production of antioxidant enzymes. The greater decrease in Pn and Tr in RNAi lines under drought stress may reflect the reduced capacity of the antioxidant system. The same trend was observed in F*v*/F*m*. Under drought stress, plant drought resistance is related to the accumulation of osmoregulatory substances such as proline. Here, we observed lower proline concentrations in RNAi lines under DS+M. This may also have contributed to the greater drought sensitivity observed in the transgenic lines.

The increased tolerance of mycorrhizal plants to drought stress is related to changes in plant hormone homeostasis. Of these changes in hormonal activity, changes in ABA signaling have been studied the most in-depth^34^. As expected, drought caused an increase in ABA, and the ABA content of mycorrhizal plants increased more than that of nonmycorrhizal plants. In addition to its importance to drought tolerance, the production of ABA is essential for the establishment and functioning of AM symbiosis^20,45^. Hence, the increased ABA levels in AM symbiosis-stressed plants served not only to improve drought tolerance but also to enhance and maintain AM symbiosis. In our study, lower ABA levels were observed in the RNAi lines due to the lower AMF colonization rate in the RNAi lines.

**Fig. 10:**
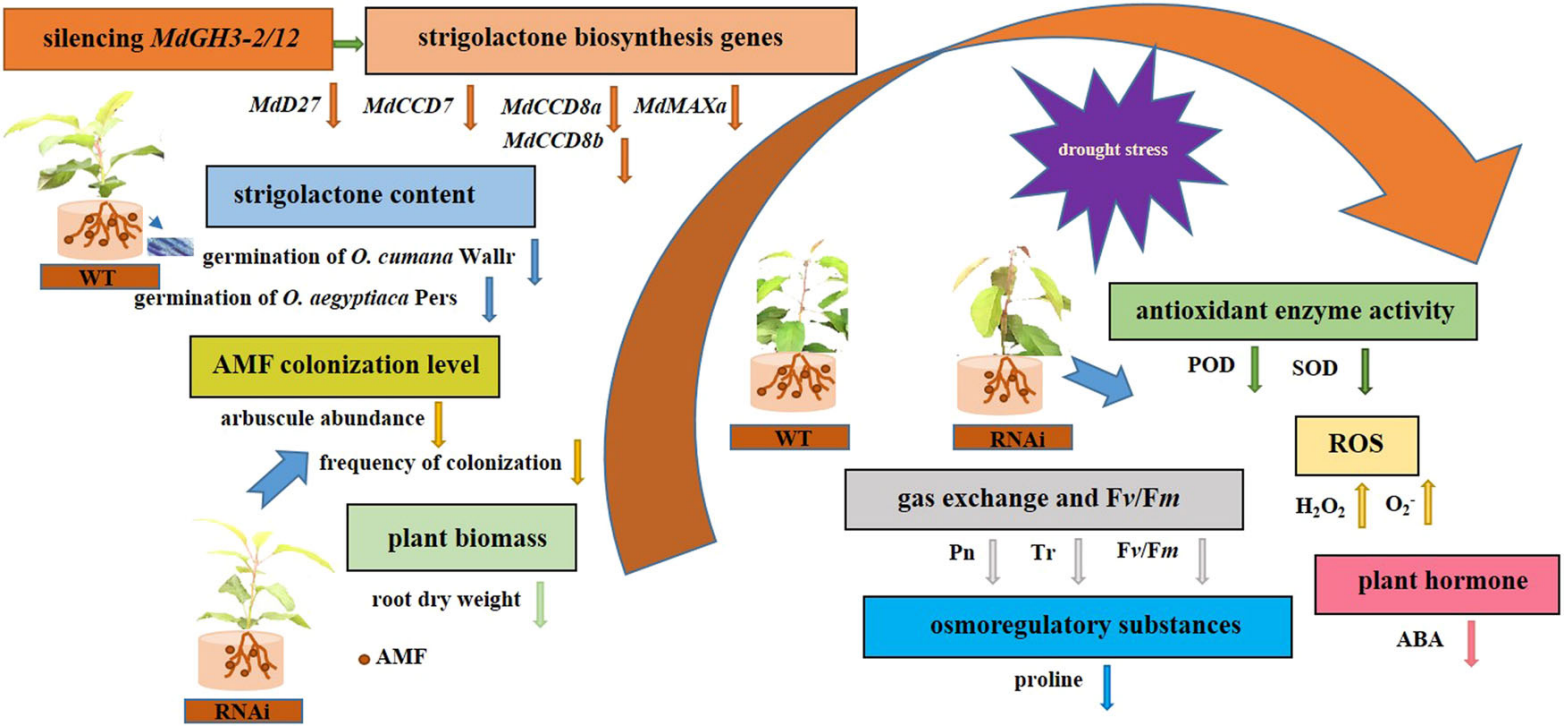
A working model for silencing *MdGH3-2/12* in response to AMF inoculation and drought in apple. *MdGH3-2/12* silencing resulted in the downregulation of *MdD27*, *MdCCD7*, *MdCCD8a*, *MdCCD8b*, and *MdMAXa*, and there was a corresponding change in root strigolactone content. Furthermore, we observed lower root dry weights in RNAi lines under AM inoculation conditions. Under drought stress, mycorrhizal transgenic plants showed higher sensitivity than WT, as indicated by higher relative electrolytic leakage and lower relative water contents, osmotic adjustment ability, ROS scavenging ability, gas exchange capacity, chlorophyll fluorescence values, and abscisic acid contents

In brief, we functionally characterized *MdGH3-2/12* by downregulating them in apple and addressed their role in AM symbiosis (Fig. 10). Although further studies are necessary to fully understand how *MdGH3-2*/*12* regulates AM symbiosis, our data demonstrate their importance for the establishment of AM symbiosis and for drought stress resistance.

## Materials and methods

### Plant material, growth conditions, and treatments

The leaves, branches, bark, flower buds, and mature flowers of ‘Royal Gala’ (*Malus domestica*) apple trees and the fruits, leaves, and roots of ‘Winter Red’, a cultivar of crabapple (*Malus* spp.), grown at the horticultural experimental station of Northwest A&F University (Yangling, Shaanxi, China) were sampled. The mature leaves of ‘Qinguan’ apple were used for gene cloning. A line with high regeneration capacity isolated from ‘Royal Gala’ (*M. domestica*) named GL-3 was used for genetic transformation^46^. After rooting on MS agar medium, the plants were transferred to small plastic pots (8 × 8 cm) that contained a substrate/perlite/vermiculite mixture (4:1:1, v/v/v) that had been sterilized by autoclaving at 121 °C for 2 h. After one month, the mycorrhization assay plants were moved to larger plastic pots (9 × 10 cm) that contained the sterilized mixture described above. Ten grams of fungal inoculum was added, and noninoculated control plants received the same amount of sterilized mycorrhizal inoculum. The AM fungus was *R. irregularis*. Two months after transplantation, AMF seedlings and non-AMF seedlings were exposed to well-watered or drought conditions, with 40 pots in each treatment. Water was withheld from the drought-stressed seedlings for 9 days.

### Genetic transformation of apple

A 185-bp fragment of *MdGH3-2* was cloned into the Gateway transfer vector pDONR222 and then into the binary vector pK7WIWG2D to create the two RNAi silencing constructs. A schematic drawing of the silencing vector is shown in Fig. S5. The *Agrobacterium*-mediated transformation of apple was performed as previously described, using GL-3 as the genetic background^44,46,47^. The primers used for constructing all vectors are shown in Table S2. A Genome Walking Kit (Takara, Dalian, China) was used to identify the transgene insertion site.

### Biomass production and observation of AM development

After 8 weeks of the NM and M treatments, the NM and M seedlings were harvested and separated into shoots and roots to measure their biomass. Growth parameters, including stem diameter and plant height, were also recorded. The root staining procedure followed the methods of Koske and Gemma^48^ and Huang et al^39^. The degree of root mycorrhizal colonization was calculated according to the method of Trouvelot et al.^49^ under an Olympus microscope.

### Subcellular localization assays in *Nicotiana benthamiana* leaves

The coding regions without stop codons of *MdGH3-2* and *MdGH3-12* were recombined into the pcambia2300-GFP vector (Fig. S5). The AtCBL1n:mCherry construct was used as a marker for plasma membrane protein localization^50^. Then, 100 mg ml^−1^ 4′,6-diamidino-2-phenylindole (DAPI, MP Bio, Santa Ana, CA) was used to locate the fluorescent proteins in the nucleus. Instantaneous transformation and fluorescence signal observation were performed as described by Liu et al.^51^.

### Germination assay

After 8 weeks of the NM treatment, the roots were placed in envelopes in a 105 °C oven for 1 h. The oven temperature was then adjusted to 80 °C, and the roots were fully dried. The supernatant obtained by centrifugation was diluted with methanol to final concentrations of 10 and 1 mg/L. Germination bioassays were performed according to Ma et al.^52^.

### Measurements of ABA content

Frozen root tissue (0.1 g) was ground into a 1 mL extraction solution containing methanol:isopropanol, 20:80 (v/v), with 1% glacial acetic acid. After standing at 4 °C for 12 h, the mixture was centrifuged at 12000 g for 5 min at 4 °C. Afterward, the supernatants were combined and passed through a 0.22-μm PTFE filter. The concentrations of ABA were determined as described by Guo et al.^53^.

### Photosynthetic characteristics and F*v*/F*m*

Gas exchange parameters, including the net photosynthetic rate (Pn) and transpiration rate (Tr), were monitored using a portable photosynthesis system (LI-6800, Li-Cor Biosciences, Lincoln, NE, USA). For each treatment, the 4th–6th leaves from the stem base were selected for measurement. F*v*/F*m* was measured using a MINI-PAM-II fluorometer (Imaging PAM, Walz, Effeltrich, Germany).

### Relative water content and relative electrolytic leakage

The RWC and REL were determined and calculated according to the method described by Sun et al.^44^ and Dionisio-Sese and Tobita^54^.

### Determination of ROS accumulation, proline, and antioxidant system activity

The levels of H_2_O_2_, superoxide radical (O_2_^-^), and proline and the activities of superoxide dismutase (SOD) and peroxidase (POD) were detected via Suzhou Comin Biotechnology test kits (Suzhou Comin Biotechnology Co., Ltd, Suzhou, China).

### RNA extraction, DNA isolation, and qRT-PCR analysis

Total RNA was extracted from apple plants via a Wolact plant RNA isolation Kit (Wolact, Hong Kong, China). Apple genomic DNA was isolated with a Wolact Plant Genomic DNA Purification Kit (Wolact, Hong Kong, China). qRT-PCR analysis was carried out as previously described by Huo et al.^55^. *Malate dehydrogenase* (*MDH*) transcription was used to normalize the levels of different genes. Table S2 lists the primers used in this study.

### Identification and phylogenetic and gene structure analyses of *GH3* genes

The protein sequences of 19 GH3 genes from *Arabidopsis* were downloaded from the NCBI database (https://www.ncbi.nlm.nih.gov) according to Xu et al.^56^. All AtGH3 proteins were used as queries in a BLASTP search against the apple genome database (https://www.rosaceae.org/species/malus/malus_x_domestica). Candidate sequences were confirmed using the Conserved Domain Database (http://www.ncbi.nlm.nih.gov/Structure/cdd/wrpsb.cgi) and SMART Database (http://smart.emblheidelberg.de/) and compared with Yuan et al.^57^. The OsGH3 protein sequences were downloaded from the NCBI database (https://www.ncbi.nlm.nih.gov) according to Jain et al.^58^. The phylogenetic trees were estimated with the MEGA5 program using the neighbor-joining method. Gene structures were constructed using TBtools (version 1.046). Sequence alignment of *MdGH3-2* and *MdGH3-12* was performed with DNAMAN (version 6.0.3.99).

### Statistical analysis

SPSS 16.0 was used for the statistical analysis. The data were subjected to independent-samples t-tests and one-way ANOVA and expressed as means ± SDs.

## Supplementary information

Supplementary materials
