# Supplementary material for: Silencing MdGH3-2/12 in apple reduces drought resistance by regulating AM colonization
Source: Hortic Res. 2021 Apr 1;8:84. doi: 10.1038/s41438-021-00524-z (PMC8012562; doi:10.1038/s41438-021-00524-z)
Supplement: Supplementary file 1 — Supplementary materials [file 41438_2021_524_MOESM1_ESM.docx]

**Table S1.** (a) Primers used for Genome Walker analysis of T-DNA integration sites. (b) The sequence data obtained revealed that Lines Ri-1and Ri-9 have different genomic DNA regions alongside the T-DNA left border.

(a)

| Name | Primers |
| --- | --- |
| LSP1 | ATATATCCTGCCACCAGCCAGC |
| LSP2 | CCGTTGTAAGGCGGCAGACTTT |
| LSP3 | GCAACTAAGCTGCCGGGTTTGAAA |

(b)

| Name of transgenic line | Sequence adjacent to the T-DNA left border |
| --- | --- |
| Ri-1 | CATAAGTAATCTTGGAACCTTCATTGCTTACTTGGGACATATAATTTCAGCTTGTGGAGTGACCGTGGGCCCCTCCAAGATAGTAACAATTTTTTAATGGCCGACTCCCTCGAGCATCTGTGCTCTCCGCGGGTTCTTGGGTCTCGCCGGTTAATACCGCAAGTTTGTCCGTCATTTTGGTCTCATTGCCAAGCCCCTCACAGACTTACTCAAGAAAGACAGCTTCTCATGGTCGCCCACAGTGAACACGGCCTTTTTGACCATCAAGGTGGCATTGTCTACAACTCCAGTCCTGGCACTCCCCGATTTCACCAACTCATTCAGCTTGGAGTGTGAAGTGTCCAACGTCGACATTGGTGTCGTGTTGTCCTAAGATAATCACCCAATTGAGTTCCTTAGCCAGCCCTTGGCACCCAAACACCAATCACTCTCAGTGTATGACAAGGAAATGCTAGCTGTGGTGTTTGCCGTCCAAAAGTGGCATTCCTGTTTAATTGGACACCACTTCACCATTCTCACGGAACACCATACTCGAAA |
| Ri-9 | CATTAGTATCTAGCTGCTCTCCCTGATTACTTGGCCTCCAACTAATTTGTATCTTGCTGAAAACTGGCTACCCTCTTATTAGGAATTGGATGATCTCACTGATGCCCTGTTAAAACCATTGATGATCAGGGGCTGTGAGATTTGAAGGCTAATACCTATTTGTTCCCCGTTATTGTTTGCTCAATTCTGGAGATGACACTGACTAAAGATATTGCTAAGGACATCTGCAATTCCTTGAGGGAGAGTTCCCTTTGCCGAACCCCACTCATGCATAATTTACTTCAAGCTCTTCTAAAGAGATTGAATTTCCACACCTGAACGAACTTTATTCTGAGATTGACAACTTCCTATGCACTCGCACCATAACCTACAAGAATATGATTCTTGGGGAAAAGATGGGAGATGTGGTGGTCATAAAAAAGATTCTCTGATCGTGGAATCCTAAATATGACTCTGTGGAGTTTCCCATTCCTTACCCTTTTTATCTTGTCCTCTTGCCCAACGATCAACACCATCACTCAACACC |

**Table S2.** Primers used in this study.

| Use | Primer name | Forward primer (5'- 3') | Reverse primer (5'- 3') |
| --- | --- | --- | --- |
| qRT-PCR | *MdGH3-1* | CGGAACCTTCGAGCTGCTGTG | TCGGATTGATGGCACTGTGATTCG |
|  | *MdGH3-2* | CAAGCCGTCCGAGGTATCTTACAC | CGACACAGTCCAGCGTAGGTTG |
|  | *MdGH3-3* | AGGCACCGCAGTTCAACTTCATC | GGATTGTTGTTGTGTCCGCATAGC |
|  | *MdGH3-4* | ATGTCAACACCTTGCGGCTTACC | AGGACTTGGTGGCGGTGGAC |
|  | *MdGH3-5* | GCCATCGACAACGCCTCCAAG | TCCGAGTTCGCCGAGTCCTTC |
|  | *MdGH3-6* | CGCCGACACGACGACAATCC | GTTAGGCAGCACTGGTCCATCAC |
|  | *MdGH3-7* | GGTTCTTCTGAAGGATGGGTTGGG | GCTGCTCACCGCTGCTGTTC |
|  | *MdGH3-8* | TCCGTGCCGCTGTCTACCTAC | TCGACTGAGAGGAGAGGCTGTTG |
|  | *MdGH3-9* | AGATGAGGATGAGGAGGTGCGTAG | TCACTTGGAGCACATCACCGATTC |
|  | *MdGH3-10* | TGTGCTGAGAGTGGCCTCCTTC | GTCCGTCTTGTCCGAGTCAATGC |
|  | *MdGH3-11* | AGGCGAAATGTGGTTCTGAGCATC | TTGAGGAGGTGTCGGCGTAGC |
|  | *MdGH3-12* | GCGAGAGTTCAACACCACCGTAG | CTGCCTTGTCTGTACACCGAGTTC |
|  | *MdGH3-13* | ACAAGAGCAGTAACTTCCGCAACC | GCTGTCTGAGCACAAGATGGTCTC |
|  | *MdGH3-14* | AACAGCAGGAACAGCCACAACTC | GAGAAGAAGAGGCCGAGGAGGAG |
|  | *MdCCD7* | GCAGCAGCAACTTCAGGTT | TGTAGCCATCATCTTCTTCAGA |
|  | *MdCCD8a* | ACACTCGGCACATCCTATCG | CGGTCCACGGCTACAATCA |
|  | *MdCCD8b* | TTGTGGCGAGTGTGGAAGT | ATTGTGGTGTCAGCGTTGTG |
|  | *MdMAXa* | CCAGAAGTCGAGAGGAAGTTGCTG | TGAAGATCATGAGCGGTTGGCATC |
|  | *MdD27* | CCTGGCTCAACCAAGGCATGTAC | GCATCTGCCGGTCTTGTGTCTG |
|  | *MdNSP1* | CAGCGAAGGCACTGACGAACC | GCTCTTCCTCCATCAATGGCATCC |
|  | *MdMDH* | CGTGATTGGGTACTTGGAAC | TGGCAAGTGACTGGGAATGA |
|  |  |  |  |
|  |  |  |  |
| *MdGH3-2/12*-RNAi | *MdGH3-2/12-RNAi* | GGGGACAAGTTTGTACAAAAAAGCAGG  CTTCGAGTCGTTGAACTCGGTG | GGGGACCACTTTGTACAAGAAAGCT  GGGTTAGAAGTTCCATGATGGGG |
| PCR identification of  transgenic apples | L1 | CCAACTTTGTACAAAAAAGCAGGCT |  |
|  | L2 | CCAACTTTGTACAAGAAAGCTGGGT |  |
| Subcellular localization | *MdGH3-2-*2300-GFP | CGAGCTCGGTACCCGGGGATCCATGGCCATTGATACAG | CCTTGCTCACATGGTGTCGACATGACGTCGTTCTGGA |
| Subcellular localization | *MdGH3-12-*2300-GFP | CGAGCTCGGTACCCGGGGATCCATGGCCGTCGATTCAG | CCTTGCTCACATGGTGTCGACACATCGTTGCTCTGGG |

**Fig. S1** Quantification of *MdGH3s* expressions via qRT-PCR in non-inoculated and inoculated apple plants with *R. irregularis* over 8 weeks. M=mycorrhizal, NM=non-mycorrhizal. Different letters indicate a significant difference between treatments at *P*<0.05 according to independent-sample t-tests. Data are expressed as the mean ± SD (n=3).


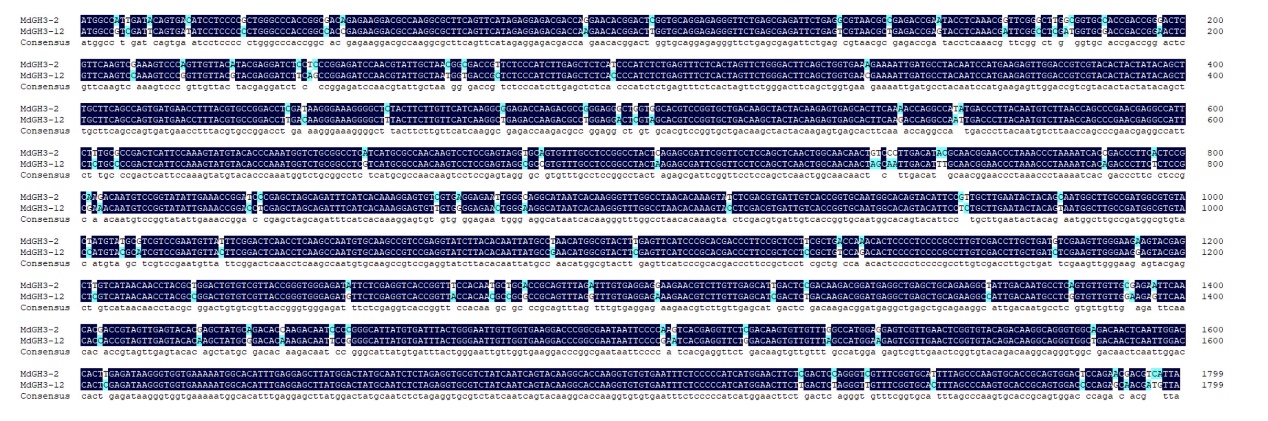


**Fig. S2** Comparison of nucleotide sequences of *MdGH3-2* and *MdGH3-12*.

**Fig. S3** Identification of transgenic lines at the DNA and RNA levels. **a** PCR with DNA; lanes: M, molecular marker DL2000; V, positive vector containing pK7-*MdGH3-2/12* plasmid; WT, nontransformed wild-type; Ri-1 and Ri-9, *MdGH3-2/12* RNAi transgenic lines. **b** qRT–PCR analysis of *MdGH3-2/12* transcripts in Ri-1 and Ri-9 lines.

**Fig.S4** *MdGH3*s expression levels in the two RNAi lines. Data are expressed as the mean ± SD (n=3).

**Fig. S5** The schematic drawing of silencing vectors of *MdGH3-2/12* (a) and overexpression vectors of *MdGH3-2* and *MdGH3-12* (b).
